# Transcription Factors-Regulated Leaf Senescence: Current Knowledge, Challenges and Approaches

**DOI:** 10.3390/ijms24119245

**Published:** 2023-05-25

**Authors:** Jie Cao, Hairong Liu, Shuya Tan, Zhonghai Li

**Affiliations:** State Key Laboratory of Tree Genetics and Breeding, College of Biological Sciences and Technology, Beijing Forestry University, Beijing 100083, China; caojie@bjfu.edu.cn (J.C.); liuhairong03@bjfu.edu.cn (H.L.); tsy20@bjfu.edu.cn (S.T.)

**Keywords:** leaf senescence, multiple regulation, transcription factor, Arabidopsis, crop

## Abstract

Leaf senescence is a complex biological process regulated at multiple levels, including chromatin remodeling, transcription, post-transcription, translation, and post-translational modifications. Transcription factors (TFs) are crucial regulators of leaf senescence, with NAC and WRKY families being the most studied. This review summarizes the progress made in understanding the regulatory roles of these families in leaf senescence in Arabidopsis and various crops such as wheat, maize, sorghum, and rice. Additionally, we review the regulatory functions of other families, such as ERF, bHLH, bZIP, and MYB. Unraveling the mechanisms of leaf senescence regulated by TFs has the potential to improve crop yield and quality through molecular breeding. While significant progress has been made in leaf senescence research in recent years, our understanding of the molecular regulatory mechanisms underlying this process is still incomplete. This review also discusses the challenges and opportunities in leaf senescence research, with suggestions for possible strategies to address them.

## 1. Introduction

Leaf senescence is a programmed and complex biological process triggered by various endogenous and environmental factors, such as nutrient deprivation, salinity, extreme temperatures, and pathogen [1,2]. It involves the coordinated dismantling of cellular components and the recycling of nutrients, ultimately leading to the programmed death of the leaf [3]. This process involves multiple levels of regulation, including chromatin level modifications, transcriptional regulation, post-transcriptional regulation, translational regulation, and post-translational regulation [4,5,6]. Manipulating leaf senescence has become an important strategy to improve crop productivity and sustainability [5,7]. Delaying the onset of senescence or prolonging the senescence process can improve nutrient remobilization, photosynthetic efficiency, and stress tolerance [2].

In recent years, significant progress has been made in understanding the molecular mechanisms underlying the regulation of leaf senescence [5,8]. Several studies have reported that epigenetic modifications of chromatin play a crucial role in regulating the timing and progression of leaf senescence [9,10,11,12]. Specifically, histone methylation and acetylation have been shown to regulate the expression of senescence-associated genes (SAGs) and thus influence the onset and progression of leaf senescence [10]. Recent studies have also identified several microRNAs (miRNAs) that regulate leaf senescence by targeting SAGs. For instance, miR156 has been shown to delay leaf senescence by targeting *SQUAMOSA PROMOTER BINDING PROTEIN-LIKE* (*SPL*) genes [13], which in turn suppress the expression of SAGs. Similarly, miR164 and miR319 have been shown to regulate leaf senescence by targeting NAC transcription factors (TFs) and TEOSINTE BRANCHED/CYCLOIDEA/PCF (TCP) TFs, respectively [14,15].

At the transcriptional level, TFs have been identified as key regulators of leaf senescence [5,8]. Over the past few years, significant progress has been made in understanding the roles of TFs in regulating leaf senescence. These TFs act through complex regulatory networks integrating multiple signaling pathways, including hormones and environmental cues [8]. Among the TF families, NAC and WRKY family genes have been extensively studied and shown to play critical roles in regulating leaf senescence [6]. The NAC family genes, including ORESARA1(ORE1)/ANAC092, AtNAP/ANAC029, JUNGBRUNNEN1 (JUB1)/ANAC042, ANAC019, ANAC055, and ANAC072, are key regulators of leaf senescence in Arabidopsis [14,16,17,18]. Overexpression of *ORE1* or *AtNAP* promotes leaf senescence, while their knockdown or knockout delays leaf senescence [14,16]. The WRKY family genes, including WRKY53, WRKY75, and WRKY42, have also been identified as important regulators of leaf senescence [19,20,21]. In addition to NAC and WRKY family genes, other TF families, such as AP2/ERF, MYB, and ARR, have also been reported to regulate leaf senescence [22]. For example, ERF4/8 and ERF34 of the AP2/ERF family have been shown to promote leaf senescence in Arabidopsis [23,24]. The MYB family gene MYBH is another positive regulator of leaf senescence in Arabidopsis [25], while OsMYB102 is a negative regulator in rice [26]. Moreover, the ARR family gene ARR2 has been reported to negatively regulate leaf senescence after phosphorylation by AHK3 [27].

Despite significant progress in understanding the molecular mechanisms underlying the regulation of leaf senescence [5], we still lack a clear understanding of its molecular regulatory mechanisms. This review aims to provide an overview of the current research on leaf senescence, especially the regulatory role of TFs. We also discuss leaf senescence research challenges and possible strategies to address them.

## 2. TFs-Regulated Leaf Senescence

### 2.1. NAC TFs-Regulated Leaf Senescence

The NAC family is a prominent group of plant-specific TFs that regulate diverse biological processes. In recent years, significant progress has been made in understanding the regulatory mechanisms of NAC TFs in leaf senescence in various plant species [28]. In Arabidopsis, numerous NAC TFs have been identified to regulate leaf senescence, including ANAC002/ATAF1 [29], ANAC016 [30], ANAC017 [31], ANAC019 [18], ANAC029/AtNAP [16], ANAC032 [32], ANAC042/JUB1 [17], ANAC046 [33], ANNAC055 [18], ANAC059/ORS1 [34], ANAC072 [18], ANAC075 [35], ANAC082 [31], ANAC083/VNI2 [36], ANAC087 [37], ANAC090 [31], and ANAC092/ORE1 [38]. Intriguingly, most of the senescence-associated NAC genes are positive regulators of leaf senescence, while only a few are negative regulators, including ANAC017, ANAC042/JUB1, ANAC075, ANAC082, ANAC083/VNI2, and ANAC090. For example, ANAC032 is a transcriptional activator that positively regulates age-dependent and stress-induced senescence in Arabidopsis by modulating reactive oxygen species (ROS) production and the expression of SAGs, including NON-YELLOWING-1 (NYE1), SENESCENCE-ASSOCIATED GENES 113 (SAG113), and SMALL AUXIN UP RNA GENES (SAUR)36/SAG201 [32]. ANAC053 promotes ROS production by binding directly to the promoters of genes encoding ROS biosynthetic enzymes during drought-induced leaf senescence, similar to ANAC032 [32]. In contrast, JUNGBRUNNEN1 (JUB1, ANAC042) is an H_2_O_2_-induced NAC TF and represents a strong negative regulator of senescence. JUB1 dampens intracellular H_2_O_2_ levels and enhances tolerance to various abiotic stresses through a gene regulatory network that involves DEHYDRATION-RESPONSIVE ELEMENT BINDING PROTEIN2A (DREB2A) [17]. One possible reason for this observation is that current studies have primarily focused on senescence-up-regulated NAC genes and have overlooked senescence-down-regulated NAC genes that may play a role in suppressing leaf senescence.

**Figure 1:**
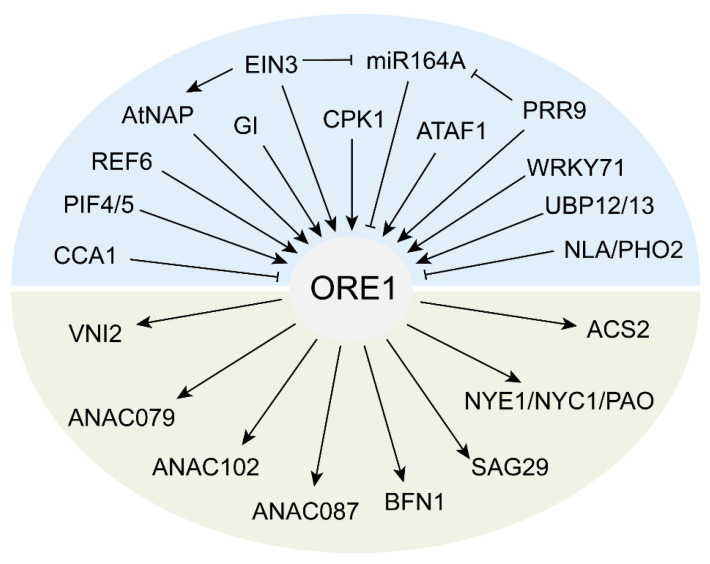
ORE1 is a hub NAC TF that positively regulates leaf senescence. ORE1 mediates leaf senescence induced by age [14], plant hormones [40,76], circadian rhythm [41,42,43], darkness [44,45], low or high ambient temperature [46,47], nitrogen deficiency [48,49], and fungi [51].

Among these NAC TFs, ORE1 is the first positive regulator of leaf senescence identified through a forward genetic screen [38], and functional deficiency can delay the process of leaf senescence induced by multiple factors [39]. Importantly, ORE1 mutants did not exhibit significant changes in other developmental processes [38], suggesting that ORE1 may be specifically involved in regulating leaf senescence. Therefore, the regulatory mechanisms of ORE1 in leaf senescence have been extensively studied and have provided valuable insights into the molecular basis of this complex process (Figure 1). Internal factors such as age and plant hormones and various environmental factors such as biotic or abiotic stresses regulate the leaf senescence process by regulating the gene expression or protein stability of ORE1 (Figure 1). The expression level of *ORE1* was up-regulated by ETHYLENE INSENSITIVE2 (EIN2) in parallel with leaf senescence but was negatively regulated by miR164. Expression of *miR164* was progressively reduced with senescence through the negative regulation of EIN2, which resulted in an elaborate up-regulation of ORE1 expression [14]. Moreover, ETHYLENE INSENSITIVE3 (EIN3), a master TF in the ethylene signal pathway, acts downstream of EIN2 to repress miR164 transcription by binding directly to the *miR164* promoter region and increases the transcriptional level of ORE1/NAC2, thereby promoting leaf senescence [40]. In addition, ORE1 mediates leaf senescence induced by circadian rhythm [41,42,43], darkness [44,45], low or high ambient temperature [46,47], nitrogen deficiency [48,49], salt stress [50], fungi [51], and reactive oxygen species (ROS) [39] (Figure 1). ORE1 promotes leaf senescence by regulating downstream target genes such as chlorophyll degradation-related genes [50,52,53,54]. Loss of ORE1 function delays the leaf senescence process induced by these factors. Recently, the crystal structure of the ORE1–NAC domain alone and its DNA-binding form have been reported [55]. This work provides a platform for understanding other plant-specific NAC protein–DNA interactions and insight into the structural basis of NAC regulators in plants of agronomic and scientific importance.

Furthermore, comparative genomic analysis of NAC genes across Arabidopsis and various crops has uncovered both conserved and species-specific regulatory mechanisms of NAC TFs in leaf senescence [56]. Notably, NAP homologs in several species, such as cotton [57], rice [58], tomato [59], bamboo [60,61], tobacco [62], and millet [63], exhibit a functionally conserved phenotype of senescence-regulated genes. ZmNAC126 was found to promote leaf senescence by regulating SAG expression in maize [64]. OsNAC2 [65] and OsNAP [58] have been shown to promote leaf senescence via ABA biosynthesis, while ONAC106 [66] and OsNAC109 [67] act as negative regulators of senescence by repressing the expression of SAG in rice. Beyond cereal crops, the regulatory mechanisms of NAC genes in leaf senescence have also been studied in other plants, such as tomatoes, trees, and roses. SlNAP2 promotes leaf senescence by regulating the expression of SAGs in tomatoes [59]. In poplar (*Populus tomentosa*), PtRD26 promotes leaf senescence by regulating the genes’ expression in chlorophyll degradation and nutrient remobilization [68]. Interestingly, age-dependent alternative splicing (AS) caused an intron retention (IR) event in the pre-mRNA encoding PtRD26. This generates a truncated protein, PtRD26^IR^, which delays leaf senescence by interacting with multiple hubs Sen-NAC TFs and inhibiting their DNA-binding activity, thus becoming a dominant negative regulator of senescence [68]. RhNAC100 regulates leaf senescence by promoting the expression of SAGs in roses [69]. The progress in research on the NAC family regulating leaf senescence has revealed the conserved and species-specific regulatory mechanisms of NAC TFs in various plant species. This knowledge can be utilized to develop strategies for regulating the leaf senescence process and improving crop yield and quality [56].

### 2.2. WRKY TFs-Regulated Leaf Senescence

WRKY genes are a family of TFs that are characterized by the presence of a conserved WRKY DNA-binding domain. In Arabidopsis, the WRKY gene family comprises 72 members involved in various biological processes. Among them, several WRKY genes have been identified as critical regulators of leaf senescence in Arabidopsis, including WRKY6 [70], WRKY22 [71], WRKY26 [72], WRKY42 [21], WRKY45 [73], WRKY53 [19], WRKY54 [74], WRKY55 [75], WRKY70 [74], WRKY71 [76], and WRKY75 [20]. Most of these WRKY TFs positively regulate leaf senescence, while WRKY54 and WRKY70 function as negative regulators by interacting independently with WRKY30 [74]. WRKY6 facilitates the leaf senescence process via binding to a receptor-like kinase *SENESCENCE-INDUCED RECEPTOR-LIKE SERINE/THREONINE-PROTEIN KINASE* (*SIRK*) gene promoter whose developmental expression is strongly explicitly induced during leaf senescence [70]. Overexpression of *WRKY22*, a direct target gene of WRKY53, has accelerated leaf senescence in Arabidopsis [71]. Similarly, WRKY45 has been found to mediate gibberellin (GA)-induced leaf senescence by interacting with the DELLA protein RGA-LIKE1 (RGL1), a repressor of the GA signaling pathway [73]. Moreover, WRKY45 directly binds the promoters of several SAGs, including SAG12, SAG13, SAG113, and SEN4 [73]. WRKY53 has a positive effect on plant senescence. It is partially involved in the SA-signaling pathway and interacts with the JA-inducible protein EPITHIOSPECIFYING SENESCENCE REGULATOR (ESR/ESP) to antagonistically regulate SA-JA signaling during leaf senescence [19]. Loss of function of WRKY75 delays age- and dark-induced leaf senescence, suggesting that WRKY75 positively affects this process. Correspondingly, gene expression of *WRKY75* gradually increases during the natural leaf senescence process [20]. WRKY75 has been shown to increase SA by directly binding to the W-box (TTGACT) sequence in the promoter of *SA INDUCTION DEFICIENT 2* (*SID2*) to activate its transcription [20]. Moreover, WRKY75 suppresses the transcription of *CATALASE 2* (*CAT2*) to repress H_2_O_2_ scavenging, which results in an accumulation of ROS levels, leading to early leaf senescence [20]. 

In addition to Arabidopsis, WRKY TFs have also been found to play crucial roles in regulating leaf senescence in various plant species. For instance, TaWRKY40-D positively regulates leaf senescence in wheat by altering the biosynthesis and signaling of JA and ABA [77]. TaWRKY42-B promotes leaf senescence by regulating *TaLOX3* gene expression, thereby enhancing JA biosynthesis [78]. In sorghum, overexpression of *SbWRKY50* delayed age-dependent and dark-induced senescence [79]. SbWRKY50 is a direct target of the key component ETHYLENE INSENSITIVE 3 in ethylene signaling but functions as a negative regulator of leaf senescence by suppressing chlorophyll catabolic pathway via directly repressing *SbNYC1* (*NON-YELLOW COLORING 1*) [79], suggesting that SbWRKY50 may act as a braking device for the signaling pathway of ethylene-induced leaf senescence. OsWRKY5 promotes leaf senescence in rice by regulating the expression of Sen-NAC TFs, including *OsNAP* and *OsNAC2* [80]. Similarly, OsWRKY42 has been shown to induce the accumulation of reactive oxygen species by directly suppressing *OsMT1d* expression, thereby accelerating leaf senescence [81]. 

In summary, WRKY genes play crucial roles in regulating leaf senescence in various plant species by regulating the expression of senescence-associated genes, which can either promote or inhibit leaf senescence. Comparative genomic analysis of WRKY genes in different species has provided insights into this important gene family’s evolution and functional diversification. Further research on the functions of WRKY genes in regulating leaf senescence will deepen our understanding of the mechanisms underlying this complex biological process.

### 2.3. ERF TFs-Regulated Leaf Senescence

ETHYLENE RESPONSE FACTOR (ERF) is one of the largest TF families in plants and plays essential roles in various physiological processes. Significant progress has been made in recent years in understanding the regulatory mechanisms of ERFs in leaf senescence. For example, AtERF4 and AtERF8 were found to accelerate leaf senescence by directly targeting ESP/ESR and suppressing its expression, leading to the activation of WRKY53 and early leaf senescence [23]. This suggests that the regulation of leaf senescence by ERFs is achieved through their interaction with other TFs or complex regulatory networks. ERF105 positively regulates freezing tolerance and cold acclimation in Arabidopsis. Overexpression of *ERF105* improved freezing tolerance, whereas the *erf105* mutant was found to be super sensitive to cold stress [82].

Interestingly, leaf senescence started earlier in *erf105* plants than in wild-type plants [82], suggesting that ERF105 integrates environmental cues into endogenous developmental signals to regulate the leaf senescence process. In contrast to ERF105, ERF34 was found to be differentially expressed under various leaf senescence-inducing conditions and negatively regulated leaf senescence induced by age, darkness, and salt stress [24]. ERF34 also promoted salt stress tolerance at different stages of the plant life cycle, such as seed germination and vegetative growth. Transcriptome analysis revealed that overexpression of *ERF34* increased the transcript levels of *COLD-REGULATED15A* (*COR15A*), *EARLY RESPONSIVE TO DEHYDRATION10* (*ERD10*), and *RESPONSIVE TO DESICCATION29A* (*RD29A*). Moreover, ERF34 directly bound to the promoters of *ERD10* and *RD29A* and activated their expression [24]. 

In addition to these findings, BrERF72 was found to be involved in the regulation of jasmonic acid (JA) production by directly activating the expressions of *BrLOX4*, *BrAOC3*, and *BrOPR3*, thereby promoting leaf senescence in Chinese flowering cabbage [83]. Meanwhile, SlERF.F5 can directly regulate the promoter activity of *ACS6* and interact with SlMYC2 to regulate tomato leaf senescence [84]. Silencing of *SlERF.F5* causes accelerated senescence induced by age, darkness, ethylene, and JA, indicating that SlERF.F5 is a negative regulator of leaf senescence in tomatoes [84]. 

In summary, ERF TFs regulate the leaf senescence by interacting with other senescence-associated TFs or integrating developmental and environmental cues. Therefore, ERF TFs are ideal candidate genes for manipulating their expression to alter leaf senescence and enhance stress tolerance, ultimately improving crop yield and quality.

### 2.4. bHLH TFs-Regulated Leaf Senescence

The basic helix-loop-helix (bHLH) TFs are crucial in regulating leaf senescence. The bHLH subgroup IIIe factors MYC2, MYC3, and MYC4 function redundantly to activate jasmonic acid (JA)-induced leaf senescence by binding to and activating the promoter of *SAG29* [85]. Conversely, the bHLH subgroup IIId factors bHLH03, bHLH13, bHLH14, and bHLH17 bind to the promoter of *SAG29* and repress its expression, thereby attenuating MYC2/MYC3/MYC4-activated JA-induced leaf senescence [85]. This antagonistic regulation by activators and repressors ensures that JA-induced leaf senescence occurs appropriately, suitable for plant survival under fluctuating environmental conditions. 

The phytochrome-interacting factors (PIFs) 3, 4, and 5, members of the bHLH TF family, have been identified as putative mediators of leaf senescence [46,86,87,88]. PIF gene mutation results in significantly enhanced leaf longevity in age-triggered and dark-induced senescence. In contrast, overexpression of these genes accelerates age-triggered and dark-induced senescence in Arabidopsis [86]. ELF3 and phytochrome B inhibit senescence by repressing PIF4/PIF5 at the transcriptional and post-translational levels. PIF4/PIF5 act in the signaling pathways of two senescence-promoting hormones, ethylene, and abscisic acid, by directly activating the expression of *EIN3*, *ABI5*, and *EEL* [88]. PIF4, PIF5, EIN3, ABI5, and EEL directly activate the expression of the significant senescence-promoting TF ORE1, forming multiple coherent feed-forward loops. These findings shed light on how classical light signaling connects to senescence in Arabidopsis [89].

ATBS1-INTERACTING FACTOR 2 (AIF2), a non-DNA-binding bHLH TF, retards dark-triggered and brassinosteroid (BR)-induced leaf senescence in Arabidopsis. AIF2 interacts with INDUCER OF CBF EXPRESSION 1 (ICE1) via their C-termini, and the AIF2-ICE1 complex up-regulates C-REPEAT BINDING FACTORs (CBFs) to negatively regulate dark- or BR-induced leaf senescence [90]. In contrast, BRASSINAZOLE RESISTANT 1 (BZR1), a positive regulator of BR signaling, suppresses *AIF2* transcript levels and accelerates leaf senescence [90]. 

MdbHLH3 regulates ethylene biosynthesis and leaf senescence by promoting the expression of dehydratase-enolase-phosphatase complex 1 (MdDEP1) in apples (*Malus domestica*) [91]. MdbHLH93 directly activates the transcription of *MdSAG18* and promotes leaf senescence in apples [92]. Interestingly, MdBT2 interacted directly with MdbHLH93 to induce its degradation via ubiquitination and delayed leaf senescence [92].

### 2.5. bZIP TFs-Regulated Leaf Senescence

The basic leucine zipper (bZIP) family is one of the largest TF families and is essential in regulating leaf senescence across different plant species. G-box-binding factor 1 (GBF1/bZIP41), a bZIP TF, is constitutively expressed in leaf tissue and initiates the onset of leaf senescence [93]. Interestingly, biochemical analysis has revealed that the threonine/serine CASEIN KINASE II (CKII) phosphorylates GBF1, negatively affecting its DNA-binding capacity to G-boxes of two direct target genes, *CAT2* and *RBSCS1a* [93]. This study explains why senescence-regulated genes expressed early in leaf development fail to induce leaf senescence due to post-translational regulatory mechanisms.

In addition, ABA-responsive element (ABRE)-binding bZIP TFs, ABF2, ABF3, and ABF4, directly regulate the expression of *NYE1/SGR1*, a key regulator of chlorophyll catabolism in diverse plant species, promoting leaf senescence [94]. For example, in litchi (*Litchi chinensis Sonn.*), LcABF1 and LcABF2 recognize ABA-responsive elements in the promoter region of *PAO* and *SGR*, enhancing their expression and accelerating leaf senescence [95]. Further research on bZIP TFs involved in regulating leaf senescence in different crops will lead to a better understanding of the molecular mechanisms underlying leaf senescence regulation.

### 2.6. MYB TFs-Regulated Leaf Senescence

The MYB family of TFs is vital in regulating plant growth and development. Several MYB genes have been identified to be involved in regulating leaf senescence. Gain- and loss-of-function analyses have indicated that MYBH is involved in the onset of leaf senescence [25]. Plants overexpressing *MYBH* exhibit premature leaf senescence, whereas *mybh-1* exhibits a delayed senescence phenotype. Biochemical analysis reveals that MYBH promotes leaf senescence, possibly by regulating auxin homeostasis [25]. In another study, MYB59 was found to inhibit SA production by directly repressing the expression of *ISOCHORISMATE SYNTHASE 1* (*ICS1*)*/SID2* and *PHENYLALANINE AMMONIA-LYASE 2* (*PAL2*) and restrain JA biosynthesis by directly suppressing the expression of *LIPOXYGENASE 2*, forming two negative feedback regulatory loops with SA and JA and ultimately delaying leaf senescence [96]. AtMYB2 regulates whole plant senescence by inhibiting cytokinin-mediated branching at late stages of development [97]. High expression of AtMYBL significantly enhances leaf senescence phenotype, with decreased chlorophyll content and higher levels of membrane ion leakage and SAG expressions [98]. OsMYB102 plays a key role in leaf senescence by down-regulating ABA accumulation and ABA signaling responses in rice [99]. OsMYB102 represses ABA accumulation by directly activating the transcription of *OsCYP707A6* and indirectly represses ABA-responsive genes such as *OsABF4* and *OsNAP* [99]. Despite MYB TFs being involved in regulating leaf senescence, there have been relatively few studies on this aspect. Transcriptome studies have identified 42 senescence-associated MYBs (Sen-MYB) in Arabidopsis [72], and it is still unclear whether they are involved in regulating leaf senescence. The relevance of these Sen-MYB TFs to leaf senescence can be revealed by generating mutants using genome editing technology.

### 2.7. Other TF Family-Regulated Leaf Senescence

The Whirly (WHY) TFs have been shown to play critical roles in regulating leaf senescence by controlling gene expression, DNA damage response, and chromatin structure. WHY1 delays leaf senescence by interacting with Histone Deacetylase 15 (HDA15) to repress the expression of *WRKY53* [100,101]. In contrast, overexpression of *WHY2* accelerates leaf senescence [102]. Growth-regulating factors (GRFs) are plant-specific TFs that have also been linked to leaf senescence. Overexpression of *rRGF3*, which is resistant to miR396, delays leaf senescence [103]. ORE15, which encodes a PLANT A/T-RICH SEQUENCE- AND ZINC-BINDING PROTEIN (PLATZ) TF, has been shown to suppress leaf senescence by modulating the GRF/GRF-INTERACTING FACTOR regulatory pathway [104]. AUXIN RESPONSE FACTOR1 (ARF1) partially regulates leaf senescence in conjunction with ARF2. The *arf2* mutant plants exhibit delayed rosette leaf senescence, while *arf1* mutations enhance the *arf2* phenotype [105]. The DNA binding-with-one-finger (Dof) proteins are plant-specific TFs closely associated with various physiological processes. Disrupting Dof2.1, a JA-inducible gene, delays dark-induced and age-dependent leaf senescence, while overexpression of *Dof2.1* promotes senescence [106]. Dof2.1 enhances leaf senescence primarily by promoting *MYC2* expression, while MYC2 also directly regulates *Dof2.1* expression [106]. Thus, Dof2.1 is an enhancer of JA-induced leaf senescence through the MYC2-Dof2.1-MYC2 feed-forward transcriptional loop. Overall, while there is growing evidence that various TFs play essential roles in regulating leaf senescence, most current studies have focused on the role of NAC in different plant species. With multi-omics approaches and genome editing technologies, the regulatory roles of more TF families in various plant species in leaf senescence will be revealed.

## 3. Challenges and Approaches

**Figure 2:**
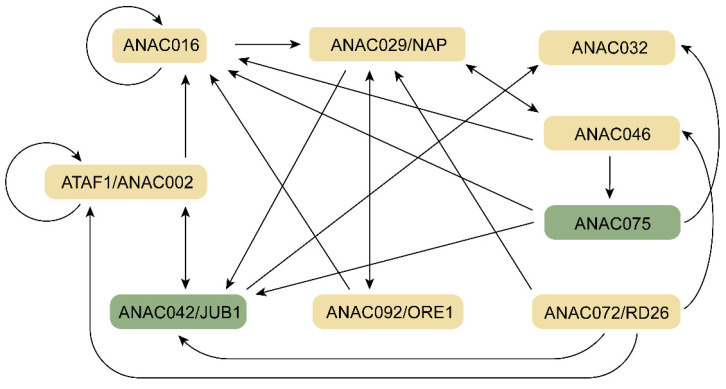
Mutual regulation among nine Sen-NAC TFs. The positive regulators of leaf senescence were labeled as yellow, and the negative regulators were labeled as green. The ChIP-Seq or DAP-Seq data were deposited in the Leaf Senescence Database as previously described [72].

Numerous regulatory genes have been identified, yet the understanding of leaf senescence remains unclear and increasingly ambiguous. How do these regulatory genes coordinate leaf senescence? Are there interactions between positive and negative regulators, and if so, do they promote or repress senescence? By analyzing ChIP-Seq or DAP-Seq results of nine NAC TFs [72], mutual regulation was found not only between positive regulators but also between positive/negative regulators (Figure 2). This partially explains why the senescence phenotype changes only slightly after the functional deletion of one gene.

In recent years, more studies on leaf senescence in crops have further confirmed the findings in Arabidopsis, indicating the conserved nature of leaf senescence regulation. However, most current studies refer to working models in Arabidopsis, replicating existing results in new species, and do not deepen the understanding of leaf senescence. Seeking model plants that are more suitable for studying senescence, such as those with shorter life cycles and smaller genomes than Arabidopsis, as well as those growing under more extreme conditions, using multi-omics and genome editing to identify regulatory genes and unravel the molecular mechanisms, may contribute to a more profound understanding of leaf senescence. We believe the problems encountered in leaf senescence research and the possible strategies to address them are as follows.

(i) Chlorophyll degradation and leaf yellowing are the most apparent phenotypic features, and *SAG12* gene expression is the most commonly used marker gene. However, these two markers are unsuitable for studying the initiation of leaf senescence. Therefore, it is urgent to find more suitable biomarkers, such as changes in metabolites (e.g., spermine) or epigenetic modifications [44].

(ii) Leaf senescence is a complex process involving a wide range of physiological, biochemical, and molecular changes, making it challenging to identify key regulators and understand their interactions. Integrating data from genomics, transcriptomics, proteomics, and metabolomics studies can help identify critical regulators and understand the complex networks underlying leaf senescence [5]. In addition, collaborative research efforts between researchers with different expertise can overcome resource limitations and increase research efficiency.

(iii) Research on how leaf senescence interacts with other plant processes, such as development, stress responses, and nutrient allocation, is lacking. More research on the interaction between leaf senescence and other plant processes is needed to gain a comprehensive understanding of the regulation of leaf senescence. Additionally, environmental factors, such as light, temperature, and nutrient availability, can significantly impact leaf senescence, but our understanding of how these factors interact with molecular regulators is limited. 

(iv) Most studies on leaf senescence have focused on Arabidopsis and a few major crops. More studies on non-model crops are needed to gain a better understanding of the molecular mechanisms underlying leaf senescence in a wider range of plants. The development of high-throughput screening methods to identify compounds that can modulate the activity of key TFs involved in leaf senescence and improve crop productivity is essential. Moreover, new technologies, such as CRISPR/Cas9 and single-cell sequencing, can provide new avenues for studying leaf senescence in various plants.

(v) Funding for research on leaf senescence is limited compared to other areas of life science. One reason for this is that the importance of leaf senescence is not yet fully understood. Studying leaf senescence not only contributes to agriculture but also provides valuable clues to human disease [107]. Increasing funding and resources for research on leaf senescence will promote the development of new technologies and investigation of new mechanisms.

## Data Availability

Not applicable.

## Abbreviations

TF	Transcription Factor
SAG	Senescence-Associated Gene
SPL	SQUAMOSA PROMOTER BINDING PROTEIN-LIKE
ORE1	ORESARA1
JUB1	JUNGBRUNNEN1
miRNA	microRNA
ERF	ETHYLENE RESPONSE FACTOR
NYE1	NON-YELLOWING-1
NYC1	NON-YELLOW COLORING 1
ROS	Reactive Oxygen Species
CAT2	CATALASE 2
COR15A	COLD-REGULATED15A
ERD10	EARLY RESPONSIVE TO DEHYDRATION10
TCP	TEOSINTE BRANCHED/CYCLOIDEA/PCF
RD29A	RESPONSIVE TO DESICCATION29A
PIF	PHYTOCHROME-INTERACTING FACTOR
AIF2	ATBS1-INTERACTING FACTOR2
ICE1	INDUCER OF CBF EXPRESSION1
CBF	C-REPEAT BINDING FACTOR
BZR1	BRASSINAZOLE RESISTANT1
bZIP	basic LEUCINE ZIPPER
bHLHSID2	basic Helix-Loop-HelixSA INDUCTION DEFICIENT2
ARF1	AUXIN RESPONSE FACTOR1
SAUR	SMALL AUXIN UP RNA GENES
DREB2A	DEHYDRATION-RESPONSIVE ELEMENT BINDING PROTEIN2A
EIN2	ETHYLENE INSENSITIVE2
SIRK	SENESCENCE-INDUCED RECEPTOR-LIKE SERINE/THREONINE-PROTEIN KINASE
RGl	RGA-LIKE1
JA	Jasmonic Acid
BR	Brassinosteroid
DEP1	Dehydratase-enolase-phosphatase complex 1
GBF1	G-box-binding factor 1
CKII	CASEIN KINASE II
ABRE	ABA-responsive element
ICS1	ISOCHORISMATE SYNTHASE 1
PAL2	PHENYLALANINE AMMONIA-LYASE 2
HDA15	Histone Deacetylase 15
PLATZ	PLANT A/T-RICH SEQUENCE- AND ZINC-BINDING PROTEIN
Dof	DNA binding-with-one-finger
